# Modeling and Control of the Redundant Parallel Adjustment Mechanism on a Deployable Antenna Panel

**DOI:** 10.3390/s16101632

**Published:** 2016-10-01

**Authors:** Lili Tian, Hong Bao, Meng Wang, Xuechao Duan

**Affiliations:** 1 The Key Laboratory of Electronic Equipment Structure Design, Ministry of Education, School of Mechanical and Electrical Engineering, Xidian University, Xi’an 710071, China; 18710849103@163.com (L.T.); xchduan@xidian.edu.cn (X.D.); 2 Collaborative Innovation Center of Information Sensing and Understanding, Xidian University, Xi’an 710071, China; 3 The Eighth Academy of China Aerospace Science and Technology Corporation, Shanghai 200233, China; tll04109030@163.com

**Keywords:** deformation adjustment, mode, Hamilton principle, LQR control

## Abstract

With the aim of developing multiple input and multiple output (MIMO) coupling systems with a redundant parallel adjustment mechanism on the deployable antenna panel, a structural control integrated design methodology is proposed in this paper. Firstly, the modal information from the finite element model of the structure of the antenna panel is extracted, and then the mathematical model is established with the Hamilton principle; Secondly, the discrete Linear Quadratic Regulator (LQR) controller is added to the model in order to control the actuators and adjust the shape of the panel. Finally, the engineering practicality of the modeling and control method based on finite element analysis simulation is verified.

## 1. Introduction

Satellites in geostationary orbit have a wide coverage area and are able to carry out a continuous observation of the area, which plays an important role in meteorological observation compared with low Earth orbit satellites. However, because of their large distance from the Earth, they need a greater antenna reflector surface area, so the deployable antenna is an eligible candidate for the restrictions of launch conditions. However, when the antenna panel is exposed to the thermal environment, it will bring about thermal deformation because of the uneven temperature field on the reflection surface structure, which will thus deteriorate the accuracy of the antenna.

However, the accuracy of the antenna reflector surface is a standard and plays an important role in the evaluation of the performance of the antenna. Additionally, it is not only closely related to the shortest wavelength of the antenna, but also has an effect on the electrical performance index, such as antenna gain, beam width, and especially the sidelobe level. Usually, the accuracy of the antenna reflector surface is related to the frequency of the work; the higher the frequency, the more stringent requirements on the surface accuracy, so the accuracy must be on the micron scale for the high frequency of operation of the 5 m antenna. Therefore, high precision reflectors and active control methods are needed to improve the accuracy of the antenna panel surface, so that the surface accuracy of large aperture large reflector antennas can achieve micron scale precision [1].

According to the literature survey, scholars use a variety of methods to adjust the accuracy of mesh antenna reflectors; Natori et al. [2] adjusted the accuracy for space very long baseline interferometry (VLBI) mission, and in [3], the shape accuracy adjustment of a flexible mesh antenna reflector by back up cable was presented. In addition, the theory of the shape accuracy adjustment of a flexible mesh antenna reflector, formula, and steps for adjustment were given for the improved mesh shape deployable antenna structure [4]. In order to adjust the accuracy of the antenna reflector in space, a piezoelectric actuator has been successfully used to compensate for the deformation panel, which is a typical electromechanical system. However, the reflector is a redundant parallel mechanism which leads to the freedom of the actuators more than the number of the degrees of freedom of the reflector, so it is difficult to control the actuator to adjust the deformation. In general, the modeling of an electromechanical system is mainly divided by the structural and control designers in the design phase; in this case, structural designers conduct the mechanical structure modeling based on a series of mechanical principles, and their contribution mainly focuses on its mechanical properties analysis, such as natural frequencies and mode shapes. Tieying Jiang [5] developed a dynamic equation of the structure based on Newton’s law and its corollary, but this model often failed to provide support in the control design method for control designers; on the other hand, control designers completed the design of electromechanical system modeling according to past experience. For example, Haiqiang Tan [6] established the force/position hybrid control model of a flexible manipulator, and designed the controller based on these models. This method is practical for simple electromechanical systems, but for complex electromechanical systems, the derived dynamical model is inconsistent with the actual model because of the lack of mechanical knowledge of the control designers, and the control method based on this model cannot achieve satisfactory results in practice.

Therefore, Gawronski [7] proposed a method of modeling and controlling by using the input–output data. Gawronski applied an open loop test of the antenna model by using a system identification procedure to record the input–output data, eventually obtaining equations of the controlled system. However, this method required experimental testing, and was not applicable in the structural design process. With the improvement of the complexity and performance requirements of electromechanical systems, the traditional method wherein structure and control are designed separately is not suitable. Consequently, some scholars have proposed modeling and controlling by using a finite element model. Firstly, the structural designers designed the structure based on the design specifications, and the controlled object model was obtained by the finite element analysis of the structure, then the control designers designed the control algorithm on the basis of the structure; for example, Gawronski [8] established the dynamic approximate model separately for 34 m and 70 m aperture antennas of the Deep Space Station (DSS) series by the finite element models of Jet Propulsion Laboratory(JPL)/Iterative Design of Antenna Structures(IDEAS), which were approximate models for the dynamics of pitch and azimuth drive and cross-coupled approximate dynamic models [8,9,10,11]. Additionally, Peter [12] built the finite element equation of the structure of the Gregor Solar Telescope, and obtained the state space equation of the telescope by extracting the first 30 order natural frequencies and modes with commercial software. These methods both built models by modes based on the structure, which can reflect the characteristics of the structure effectively, and the model could have a high accuracy. However, these modeling methods are used in single input–single output systems, and are limited for multiple input and multiple output (MIMO) systems.

In this paper, a method of structural control integrated design is proposed for a large antenna reflector electromechanical adjustment system. Firstly, the mathematical model of the reflector antenna adjustment system is established based on Hamilton principle; then, the MIMO control law is designed by the Linear Quadratic Regulator (LQR) on the basis of the model; finally, the accuracy of the model and the feasibility of the control algorithm through the simulation analysis are verified, and the design of the electromechanical adjustment system is eventually realized.

## 2. Materials and Methods

**Figure 1:**
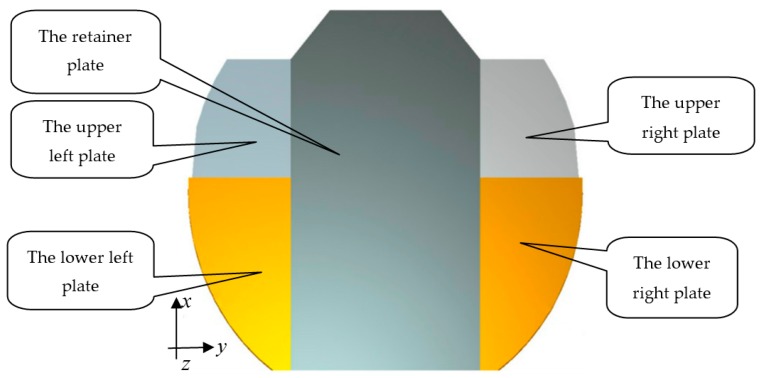
Schematic diagram of the deployed antenna.

**Figure 2:**
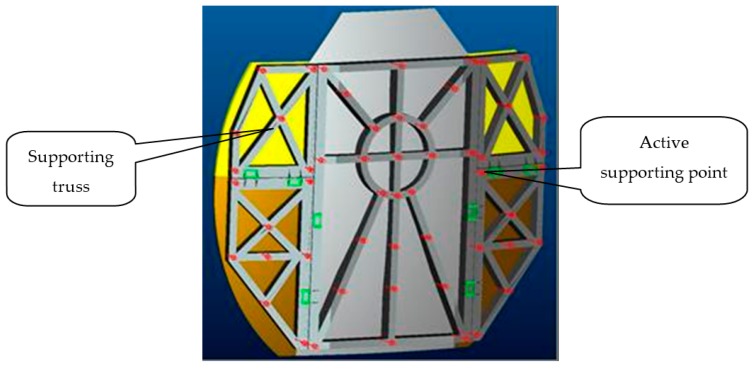
Truss structure of the antenna panel.

A 5 m diameter deployable antenna panel structure is shown in Figure 1, and the structure is composed of a retainer plate, an upper left plate, a lower left plate, an upper right plate, and a lower right plate. The truss structure of the antenna panel is shown in Figure 2, and it is composed of the main reflector and the supporting truss, where the main reflector is an integrated forming structure which is a composite sandwich structure of carbon fiber panels and aluminum honeycomb. The upper and lower panels are made of T300 carbon fiber. The specifications of the Honeycomb core are 4 mm × 0.03 mm, and the reflector thickness is 70 mm. The supporting truss is connected through the expansion mechanism, and the panel is locked after being expanded by the locking device. The support truss is mainly made of high-modulus carbon fiber named M55J, and the cross-section is 150 mm × 150 mm.

There are many active supporting points on the antenna panel truss for the piezoelectric actuator mounting point, which is used for shape adjustment of the antenna reflector; of note is that there are dozens of actuators on the reflector to adjust the shape of the panel, as in [13], so that it has dozens of degrees of freedom.

### 2.1. Modeling Based on Hamilton Principle

It is assumed that the antenna reflector is free to move, and this problem is solved by using the method of separation of variables, so:

(1) V(x,y,t)=ψ(x,y)q(t)

where ψ(x,y) is a function only related to the location of x and y in Figure 1, which indicate the vertical and horizontal coordinates of a point on the panel, respectively. q(t) is a function only related to the time t.

When the antenna reflector is deformed, the deflection surface equation is:

(2) $$w(x,y,t)=\sum_{i=1}^{N}\psi_{i}(x,y)q_{i}(t)$$

where $\psi_{i}(x,y)$ is the ith modal function of the coordinates (x,y), and $q_{i}(t)$ is the ith modal coordinates of the moment t.

The antenna is deformed under the influence of the temperature field, and the adjustment process is ignored; therefore, the final state is still in the static state, so the kinetic energy is zero.

The potential energy of the antenna reflector panel is

(3) $$P=\frac{D}{2}\int_{\Omega }\left[{\left(\frac{\partial^{2}\omega }{\partial x^{2}}\right)}^{2}+{\left(\frac{\partial^{2}\omega }{\partial y^{2}}\right)}^{2}+2\mu \left(\frac{\partial^{2}\omega }{\partial x^{2}}\frac{\partial^{2}\omega }{\partial y^{2}}\right)+2(1-\mu ){\left(\frac{\partial^{2}\omega }{\partial x\partial y}\right)}^{2}\right]d\Omega$$

where D is the bending stiffness, μ is Poisson’s ratio, ω is the curved surface deflection at this time, and Ω is the reflection panel surface area.

At this time, the generalized virtual work of the antenna reflector is:

(4) $$W=\tau w(x_{0},y_{0},t)=\tau \sum_{i=1}^{N}\psi_{i}(x_{0},y_{0})q_{i}(t)$$

In Equation (4), τ is the motor input, $q_{i}(t)$ is the ith modal coordinates of the time t, and $\psi_{i}(x_{0},y_{0})$ is the ith modal function of the coordinate $\left(x_{0},y_{0}\right)$.

Ignoring the kinetic energy of the system [14]:

(5) Δ=T−P+W=−P+W

Using a variational method [15] for Equation (5):

(6) $$\int_{t_{0}}^{t_{f}}\delta \Delta dt=0$$

According to Hamilton principle, one can obtain the following equation:

(7) $$\int_{t_{0}}^{t_{f}}\left[\sum_{i=1}^{N}\sum_{j=1}^{N}q_{j}k(i,j)-\tau \sum_{i=1}^{N}\psi_{i}(x_{0},y_{0})\right]\delta q_{i}dt=0$$

From Equation (7), one can derive the following equation:

(8) $$\sum_{j=1}^{N}q_{j}k(i,j)-\tau \psi_{i}(x_{0},y_{0})=0$$

By rewriting Equation (8) in Matrix forms, one can obtain:

(9) KX=Bτ

So far, one can derive the relationship between external force applied to the antenna reflector and the displacement of the corresponding actuating point.

Where $X={{\left[q_{1},q_{2},q_{3},\cdot \cdot \cdot ,q_{N}\right]}^{T}}_{\left(1,N\right)}$ is the modal displacement corresponding to the first of the Nth points, $\tau =\left(\tau_{1},\tau_{2}\ldots \tau_{S}\right)$ is the input force of the external motor on the S points, K is the stiffness matrix of N×N dimension, and B is the input matrix of N×S dimension corresponding to the S inputs,

$$B={\left[\begin{array}{cccc} \psi_{1}(x_{1,}y_{1}) & \psi_{1}(x_{2,}y_{2}) & ... & \psi_{1}(x_{S,}y_{S})\\ \psi_{2}(x_{1,}y_{1}) & \psi_{2}(x_{2,}y_{2}) & ... & \psi_{2}(x_{S,}y_{S})\\ \vdots & \vdots & \vdots & \vdots \\ \psi_{N}(x_{1,}y_{1}) & \psi_{N}(x_{2,}y_{2}) & ... & \psi_{N}(x_{S}y_{S}) \end{array}\right]}_{\left(N\times S\right)}$$

Thus, the stiffness of the antenna reflector surface of the ith row and the jth column can be obtained by:

(10) $$k(i,j)=D\int_{\Omega }\left[\frac{\partial^{2}\psi_{i}}{\partial x^{2}}\cdot \frac{\partial^{2}\psi_{j}}{\partial x^{2}}+\frac{\partial^{2}\psi_{i}}{\partial y^{2}}\cdot \frac{\partial^{2}\psi_{j}}{\partial y^{2}}+\mu \left(\frac{\partial^{2}\psi_{i}}{\partial x^{2}}\cdot \frac{\partial^{2}\psi_{j}}{\partial y^{2}}+\frac{\partial^{2}\psi_{i}}{\partial y^{2}}\cdot \frac{\partial^{2}\psi_{j}}{\partial x^{2}}\right)+2(1-\mu )\frac{\partial^{2}\psi_{i}}{\partial x\partial y}\cdot \frac{\partial^{2}\psi_{j}}{\partial x\partial y}\right]d\Omega$$

So, the stiffness matrix of the antenna reflector surface is obtained below:

$$K={\left[\begin{array}{cccc} k(1,1) & k(1,2) & ... & k(1,N)\\ k(2,1) & k(2,2) & ... & k(2,N)\\ \vdots & \vdots & \vdots & \vdots \\ k(N,1) & k(N,2) & ... & k(N,N) \end{array}\right]}_{N\times N}$$

Additionally, X is known as the state coordinate, assuming that Y is the actual physical coordinate, so:

(11) Y=CX

where C is the output matrix of dimension R×N, corresponding to the *R* outputs,

$$C={\left[\begin{array}{cccc} \psi_{1}(x_{1,}y_{1}) & \psi_{2}(x_{1,}y_{1}) & ... & \psi_{N}(x_{1,}y_{1})\\ \psi_{1}(x_{2,}y_{2}) & \psi_{2}(x_{2,}y_{2}) & ... & \psi_{N}(x_{2,}y_{2})\\ \vdots & \vdots & \vdots & \vdots \\ \psi_{1}(x_{R,}y_{R}) & \psi_{2}(x_{R,}y_{R}) & ... & \psi_{N}(x_{R,}y_{R}) \end{array}\right]}_{R\times N}$$

By virtue of Equations (15) and (17), we can get the deformation equation of the *S* inputs and *R* outputs of the antenna reflector:

(12) $$Y=CK^{-1}B\tau$$

Based on the above theoretical method, the deformation Equation (13) of the antenna reflector is derived, and the transfer matrix of the external force and the actual displacement is obtained:

(13) $$Z=CK^{-1}B$$

Therefore, the displacement after the ith adjustment of the antenna reflector is:

(14) Y(k+1)=Y(k)+Z*τ(k)

Among them, Y(k) is the displacement before the kth adjustment, and τ(k) is the external force of the kth adjustment.

According to Equation (13) and the matrix composition of this equation, the modal coordinates and the mode shape function of the reflection panel are needed before one can get the reflecting surface model, and all above matrices we could get from finite element analysis.

### 2.2. Modal Analysis

In order to obtain the modal coordinates and mode shape function of the reflector, we first need to select the modes. Once we obtain a series of modes after modal analysis by the finite element software, it usually contains some modes with little influence on the deformation, which could be ignored. So, in order to obtain a simple antenna reflector deformation control equation, we select the mode which reflects the bending or torsion of the reflector antenna surface deformation as the target mode.

**Figure 3:**
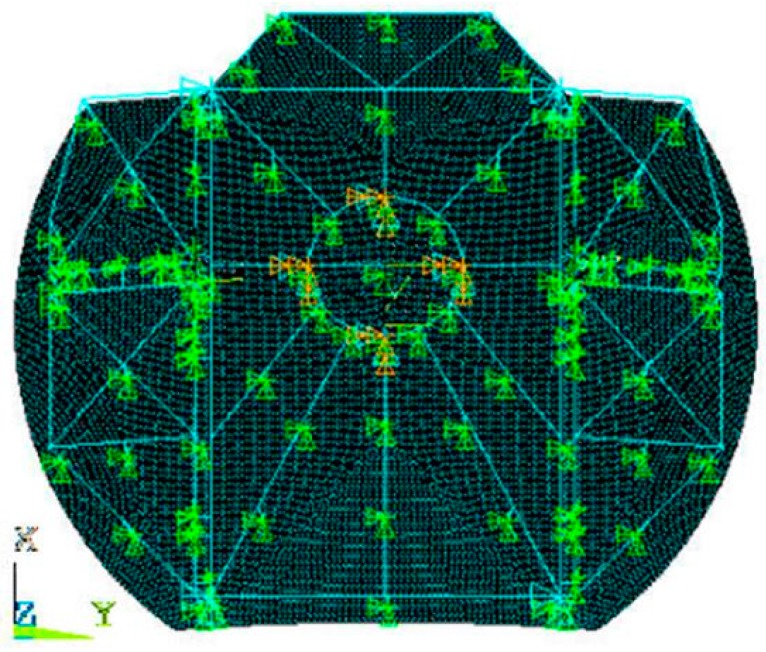
The finite element model of the antenna reflector.

The finite element model of the antenna reflector is shown in Figure 3. The model is built with a triangular element, and the main reflector is made of SHELL181 which is suitable for the analysis of thin or medium shell structures, and the supporting truss is made of BEAM188 which is suitable for the analysis of slender beams or moderately deep beams. The nodes on the four corners of the retainer plate and the center circle are fully constrained in all directions, and the other support points are constrained in the *x* and *y* directions.

We could obtain the mode shapes and natural frequencies of the antenna panel by commercial software. Taking the vertical and horizontal coordinates *x*,*y* as physical coordinates, and the antenna panel deformation *z* on the point of coordinates (*x*,*y*) as the normalized mode shape coordinates, we can obtain the modal coordinates of each point on the surface of each modal order. Then, we can obtain the fitting surface and the corresponding fitting function by the surface fitting function of the mathematics software.

**Figure 4:**
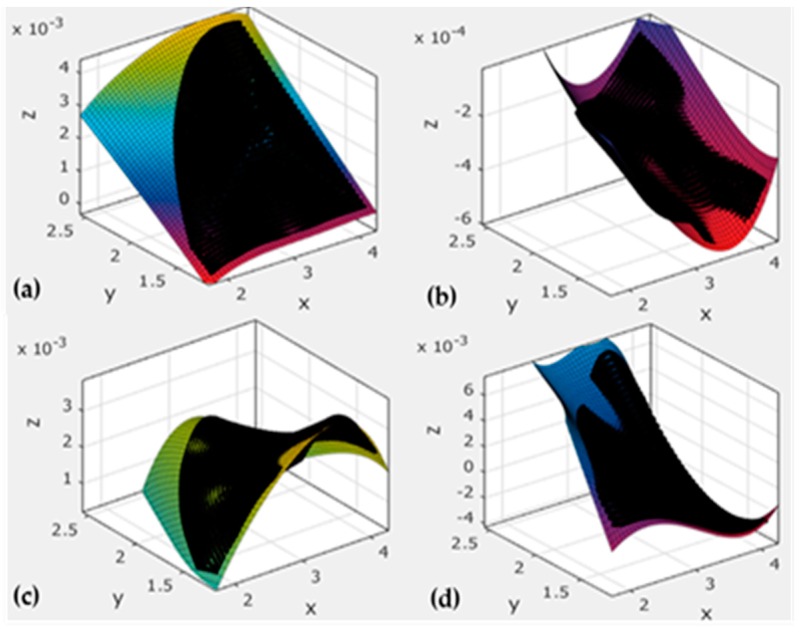
Fitting surface that is bending or torsional of the lower left plate: (**a**) is the first order mode shape that is bending; (**b**) is the fifth order mode shape that is bending and torsional; (**c**) is the sixth order mode shape that is bending; (**d**) is the tenth order mode shape that is bending and torsional.

After obtaining the fitting surface and the corresponding surface function, we selected a modal surface which was twisting or bending according to the comparison chart of each order surface fitting, and deleted the orders that had a translation or repeat; eventually we selected eight order modes 1, 2, 5, 6, 8, 9, 10, 12 as mode shape functions to calculate the stiffness matrix for the lower left plate, and then we obtained the results as shown in Figure 4.

The first order mode shape function $\psi_{1}(x,y)$ can be obtained, and similarly, the ith order mode shape function $\psi_{i}(x,y)$ can also be obtained. Eventually, we could get the model of the adjustment of the antenna reflector shape by using the stiffness matrix and Equation (14) above.

Moreover, the actuator is added to the reflective surface model. The piezoelectric ceramic actuator is used as the reflective surface. By taking the stack type piezoelectric ceramics as an example, we are able to obtain the relationship between the input voltage and the output displacement by static and dynamic experimental studies of the actuator [16]:

(15) $$\Delta x=nd_{33}V$$

where Δx is the output displacement of the actuator, n is the number of piezoelectric ceramic sheets which form a laminated piezoelectric stack, $d_{33}$ is the piezoelectric strain coefficient of the piezoelectric ceramic chip in the polarization direction, and V is the input voltage.

The application of a piezoelectric actuator in active vibration control is studied in [17], and the relationship between the input voltage and the actuator output force is obtained as:

(16) $$\tau =K_{T}nd_{33}V$$

where τ is the output force of the actuator and $K_{T}$ is the equivalent stiffness matrix of the piezoelectric ceramic stack.

The relationship between the required displacement of the actuator and the output force of the actuator is:

(17) $$\tau =K_{T}\Delta x$$

Equation (17) can be used within Equation (14), and the dynamic relationship between the input force and output displacement of the panel is then:

(18) Y(k+1)=Y(k)+Zτ(k)

where Y(k+1) is the displacement after the kth adjustment, v is the displacement before the kth adjustment, Z is the transfer matrix of the external force and the actual displacement, and τ(k) is the external force of the kth adjustment.

## 3. Controller Design

The adjustment system of the panel is a typical MIMO system, and there is a high degree of coupling between the systems. Generally, for a multivariable coupled system, the traditional design method is to design each single variable controller for each individual channel, and then a multivariable controller is composed of these controllers; however, because of the strong coupling relationship of the system, the controller designed in the traditional method cannot meet the control indexes. Therefore, the discrete LQR controller [18] is designed to control the system.

According to the transfer matrix model Equation (14) obtained in the last section, the expression of the state space expression of the plant can be obtained as follows:

(19) $$\left\{ \begin{array}{l} X\left(k+1\right)=X\left(k\right)+ZU\left(k\right)\\ Y\left(k\right)=X\left(k\right) \end{array}\right.$$

where $X\left(k\right)={\left[X_{1}\left(k\right),X_{2}\left(k\right),\cdots X_{N}\left(k\right)\right]}^{T}$ represents N states, and Z is the transfer matrix of the external force and the actual displacement. The linear quadratic optimal control of discrete systems is a method to search for the control law of the state feedback, which causes the quadratic function to take the minimum value. According to the discrete system shown in Equation (19), the quadratic objective function is selected as below:

(20) $$J=\sum_{k=0}^{\infty }\left(X^{T}\left(k\right)QX\left(k\right)+U^{T}\left(k\right)RU\left(k\right)\right)$$

where Q and R are the weight matrices of the state variables and the control variables, which are invariable real symmetry positive definite matrices. 

By the maximum principle, the linear quadratic optimal control law is obtained:

(21) $$U\left(k\right)=-{\left(B^{T}SB+R\right)}^{-1}B^{T}SX\left(k\right)=-KX\left(k\right)$$

where S is the solution of the Riccati equation, and the algebraic Riccati equation is shown as:

(22) $$S-I^{T}S+I^{T}SB{\left(B^{T}SB+R\right)}^{-1}B^{T}S-Q=0$$

According to the discrete control model shown in Equation (21), the state weight matrix is Q, and the control weight matrix R is obtained, so the discrete linear quadratic optimal control law can be obtained as:

(23) U(k)=−KX(k)

where K is the state feedback matrix to be determined.

## 4. Simulations

**Figure 5:**
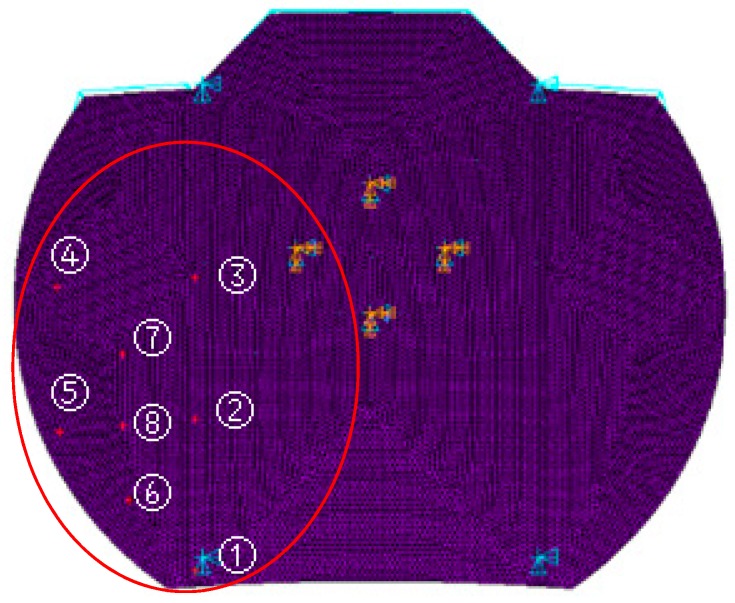
Forced simulation of the ANSYS model.

According to the modeling method of Section 2, the lower left plate is shown as an example in Figure 5. Furthermore, by distributing eight actuators on nodes 1–8 on the panel used for adjusting the plate, the 8 × 8 model Z was established as shown in Table 1, corresponding to nodes 1–8. Where $Z_{ij}$ is an influence factor of node i on node j.

In order to verify the accuracy of the model, a verification via numerical simulation is needed. At first, external force is applied to the model on nodes 1–8 in several conditions, and then the output displacement $Y_{1}$ of the model could be calculated by the approximate input and output model in Equation (14). At the same time, the same external force is applied on nodes 1–8 in the same conditions as in commercial finite element code ANSYS (Figure 5), so we could read the output displacement $Y_{2}$ of the corresponding eight nodes through the simulation analysis. Finally, the results of comparing $Y_{1}$ and $Y_{2}$ under several conditions are shown in Table 2.

**Figure 6:**
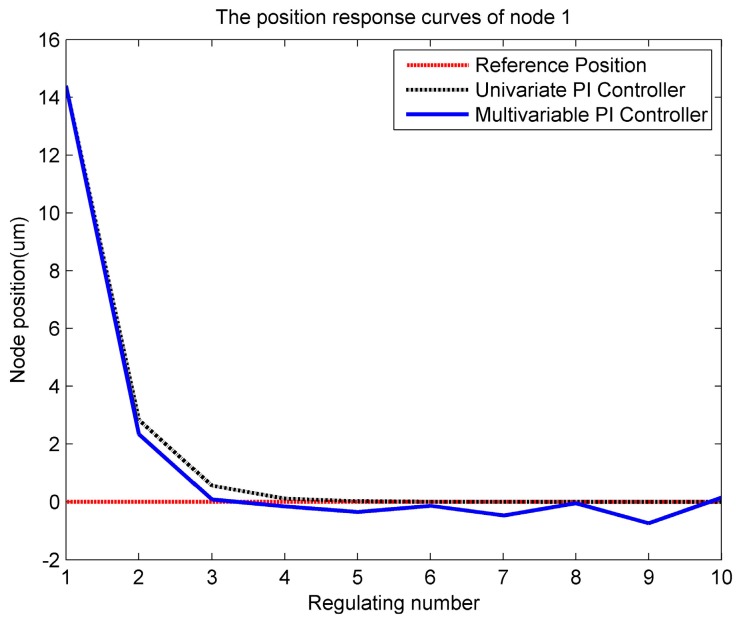
The position adjustment curve of node 1 by the Proportion-Integral (PI) controller.

By comparing the results, we see that the two groups of data are obviously different, but the changing trends of the two sets of data are the same. Therefore, we can draw the conclusion that the approximate model established is able to reflect the structural properties of the ANSYS model, and it can be used to design the controller and the control algorithm. After obtaining the mathematical model, we firstly design a univariable Proportion-Integral (PI) controller and a multivariable PI controller with the traditional control method; taking node 1 as an example in Figure 6, we can see that node 1 could reach the reference position after adjustment by the univariable PI controller, but could not reach it by the multivariable PI controller. So, we propose the use of a LQR controller.

According to the discrete control model of the lower left plate shown in Equation (19), we obtain Q and R by repeated trial and error, and take *Q* = *diag*(5000,2000,2000,10000,10000,15000,5000,50000) as the state weighted matrix, and *R* = *diag*(1,1,1,1,1,1,1,1) as the control weighted matrix. Therefore, we could obtain the feedback matrix K of the eight actuators for the lower left plate according to Equation (23), as shown in Table 3, $K_{ij}$ is an feedback factor of node i on node j.

By taking the temperature deformation of a moment in the vernal equinox as the initial displacement, using the LQR control algorithm, calculating the input force, and finally applying the force into the corresponding nodes of the ANSYS model, the results of adjusting each node can be obtained as shown in Figure 6.

**Figure 7:**
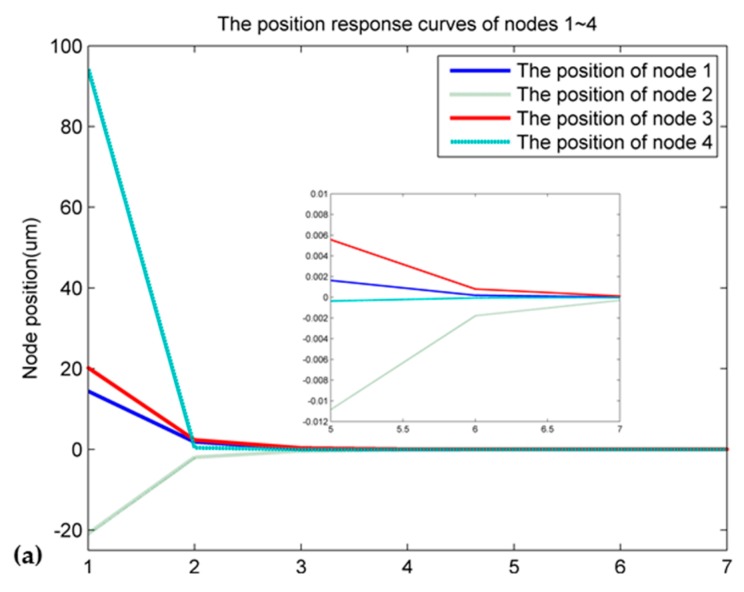

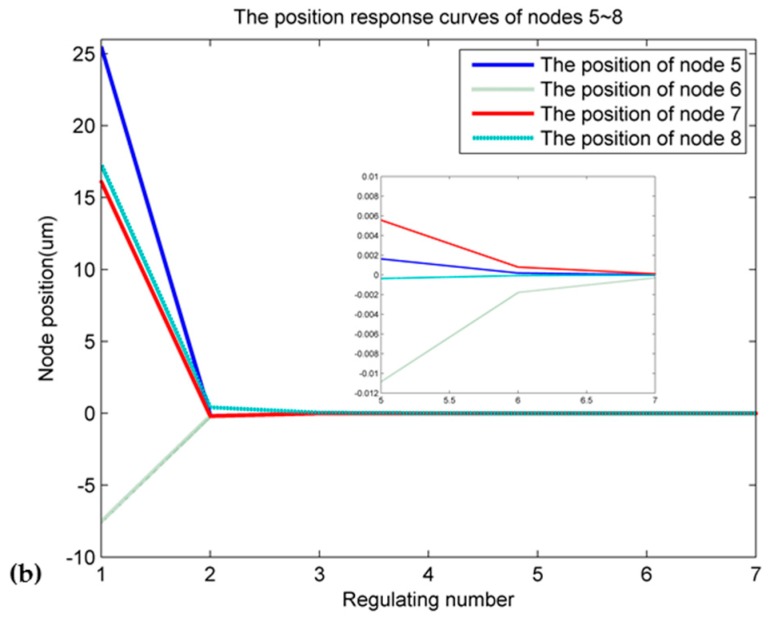
The position adjustment curve of nodes 1–8: (**a**) the position response curves of nodes 1–4; (**b**) the position response curves of nodes 5–8.

It can be seen in Figure 7 that the initial displacements of the eight nodes are far away from zero, and the maximum displacement is more than 90 μm. However, after adjustment, the displacements in the second regulating decreased obviously, and the eight nodes almost reached the target surface after 5–8 adjustments. At the same time, we extracted the node coordinates from ANSYS and calculated the RMS for the eight nodes. The RMS is 37.5 μm before the adjustment, and it decreased to 5.9 μm after the adjustment, therefore reducing the RMS by 84.3%. On the other hand, the RMS for the lower left panel before the adjustment was 52.7 μm, and it decreased to 24.2 μm after the adjustment, so the RMS was reduced by 54% on the lower left panel. Additionally, we know that the approximate model established is able to represent the relationship between the input force and the output displacement of the actual model. The difference between the approximate model and the ANSYS model does not affect the design of the controller.

## 5. Conclusions

In this paper, a method for the integrated design of structure and control is proposed for deformation; this method reduced the structure information greatly, and it strengthened the exchange of the structure and the controller, so that the information of the structure could contribute to the control portion of the design. And the structure could be improved by the feedback information that affects the performance of the structure. Finally, the ANSYS simulation experiment indicates that this method of structural control integration is effective.

## Figures and Tables

**Table 1:** Transfer matrix of the lower left plate.

$Z_{ij}$	1	2	3	4	5	6	7	8
**1**	0.0525	0.0084	−0.0054	−0.0110	0.0109	0.0282	0.0003	0.0103
**2**	0.0159	0.1496	0.0767	0.1016	0.1156	0.0767	0.1149	0.1272
**3**	−0.0066	0.0679	0.1310	0.1565	0.1099	0.0529	0.1193	0.0904
**4**	−0.0377	0.0674	0.1311	1.2854	1.1341	0.5207	0.6880	0.6321
**5**	−0.0009	0.0963	0.0993	1.1489	1.2822	0.6277	0.6924	0.7184
**6**	0.0287	0.0696	0.0546	0.5478	0.6400	0.4287	0.3442	0.3723
**7**	−0.0044	0.1027	0.1159	0.7100	0.6995	0.3391	0.4702	0.4207
**8**	0.0083	0.1177	0.0897	0.6568	0.7283	0.3698	0.4233	0.4854

**Table 2:** Comparison of the approximate model and the ANSYS model.

Forced Number	Force (N)	Approximate Model (mm)	ANSYS Model (mm)
1	1000	0.1300	0.07527
2	−500	0.0512	0.11062
3	1000	0.3795	0.25606
4	−500	0.1831	0.06539
5	1000	1.0748	0.85711
6	−500	0.9403	0.80501
7	1000	0.5097	0.52377
8	−500	0.5132	0.46679

**Table 3:** Feedback matrix *K* of the eight actuators for the lower left plate.

$K_{ij}$	1	2	3	4	5	6	7	8
**1**	18.7700	−1.1285	0.5362	0.8827	0.8241	−3.5113	0.2569	−0.3165
**2**	−1.0920	12.8232	−3.4966	2.1748	3.3688	−0.5431	−4.3024	−6.2003
**3**	0.4759	−3.3923	11.8982	−1.0247	2.8461	0.0475	−4.6879	−0.3009
**4**	0.8858	2.2394	−0.9862	5.7688	−2.6642	0.5768	−5.7366	0.3834
**5**	0.8321	3.7598	2.8797	−2.6517	12.1776	−4.8199	−4.6021	−8.3840
**6**	−3.5954	−0.5427	−0.0066	0.5836	−4.8108	9.8095	0.2140	−1.0532
**7**	0.2784	−4.5508	−5.0232	−5.7180	−4.5889	0.2099	22.1431	−2.8526
**8**	−0.2797	−6.7561	−0.0268	0.3542	−8.3148	−1.0323	−2.9019	18.9238
